# PUB11-Dependent Ubiquitination of the Phospholipid Flippase ALA10 Modifies ALA10 Localization and Affects the Pool of Linolenic Phosphatidylcholine

**DOI:** 10.3389/fpls.2020.01070

**Published:** 2020-07-15

**Authors:** Juliette Salvaing, César Botella, Catherine Albrieux, Valérie Gros, Maryse A. Block, Juliette Jouhet

**Affiliations:** Univ. Grenoble Alpes, INRAE, CNRS, CEA, IRIG, LPCV, Grenoble, France

**Keywords:** glycerolipid, flippase, ubiquitination, membrane domain, lipid trafficking

## Abstract

Biogenesis of photosynthetic membranes depends on galactolipid synthesis, which relies on several cell compartments, notably the endoplasmic reticulum (ER) and the chloroplast envelope. Galactolipid synthesis involves lipid trafficking between both membrane compartments. In *Arabidopsis*, ALA10, a phospholipid flippase of the P_4_ type-ATPase family, counteracts the limitation of monogalactosyldiacylglycerol (MGDG) production and has a positive effect on leaf development. ALA10 locates in distinct domains of the ER depending on the ALIS (ALA interacting subunit) subunit it interacts with: close to the plasma membrane with ALIS1, or next to chloroplasts with ALIS5. It interacts with FAD2 (Fatty acid desaturase 2) and prevents accumulation of linolenic (18:3) containing phosphatidylcholine (PC) stimulating an increase of MGDG synthesis. Here we report that ALA10 interacts with PUB11 (plant U-box type 11), an E3 protein ubiquitin ligase, *in vitro* and *in vivo*. ALA10 is however ubiquitinated and degraded by the 26S proteasome in a PUB11-independent process. In *pub11* null mutant, the proteasome-dependent degradation of ALA10 is retained and ALA10 is still subject to ubiquitination although its ubiquitination profile appears different. In the absence of PUB11, ALA10 is constrained to the ER close to chloroplasts, which is the usual location when ALA10 is overexpressed. Additionally, in this condition, the decrease of 18:3 containing PC is no longer observed. Taken together these results suggest, that ALA10 contributes in chloroplast-distal ER interacting domains, to reduce the 18:3 desaturation of PC and that PUB11 is involved in reconditioning of ALA10 from chloroplast-proximal to chloroplast-distal ER interacting domains.

## Introduction

Each membrane of plant cells has a specific glycerolipid composition. Like in other eukaryotic organisms, a high proportion of phospholipids is present in the endomembrane network and in mitochondria with a prevalence of phosphatidylcholine (PC). For photosynthetic function, in chloroplasts, plant cells have also an important network of membranes and the chloroplast membranes show only a low proportion of phospholipids and a specific enrichment in non-phosphorylated galactolipids with up to 50% of monogalactosyldiacylglycerol (MGDG) (Block et al, 1983). The lipid composition of each membrane compartment is however sensitive to fluctuation of developmental and environmental stimuli (Sandelius and Liljenberg, 1982; Jouhet et al., 2007; Zheng et al., 2016). Moreover, the lipid composition is not homogenous laterally and transversally along membranes, displaying domain-specific dissimilarities with possible correlation between the organization of these domains and their role in response to distinct stimuli (Gronnier et al., 2018).

In plants, glycerolipids are primarily synthesized in the ER and in chloroplasts. Their synthesis is fed by the production of fatty acids (FA) in chloroplasts. Galactosylation of diacylglycerol (DAG) by the MGDG synthase MGD1 in the inner membrane of the chloroplast envelope gives rise to thylakoid MGDG (Boudiere et al., 2014). MGD1 is activated by phosphatidic acid (PA) from diverse origins, notably coming from phospholipase-mediated hydrolysis of extraplastidial PC (Dubots et al., 2010). In addition, MGD1 is supplied with its DAG substrates either generated from *de novo* synthesis in the chloroplast (the prokaryotic pathway), or derived from linoleic (18:2) containing PC of ER origin (the eukaryotic pathway). In PC, linoleate results from the desaturation of oleate (18:1) by Fatty acid desaturase 2 (FAD2) (Karki et al., 2019; Ohlrogge and Browse, 1995). Preservation of a pool of 18:2 containing PC suitable for MGDG synthesis is therefore dependent on the overall FA metabolism, i.e. FA synthesis in chloroplasts, FA export from chloroplasts and FA desaturation in the ER.

In leaves, diurnal oscillation of the overall FA composition was observed with an increase of oleic acid during the day and linolenic acid (18:3) during the dark period (Browse et al., 1981). Several steps of regulation are likely involved in diurnal oscillation of 18:1/18:3 lipids. The first one is the light/dark modulation of FA synthesis in chloroplasts due to light enhancement of acetyl-CoA carboxylase (ACCase) which altogether results in coordination of FA synthesis with photosynthesis (Sasaki et al., 1997). This however does not explain the increase of desaturated over saturated lipids during the dark period unless there is a limitation of desaturation during the day (Mei et al., 2015).

ALA10 has been previously identified as a modulator of the MGDG/PC ratio in leaves (Botella et al., 2016). Upon chemical inhibition of MGD enzymes by Galvestine-1, a strong enhancement in expression of ALA10 was observed suggesting a link between this protein and regulation of MGDG formation (Botte et al., 2011). Moreover, ALA10 is an ER phospholipid flippase of the P_4_ type-ATPase family that interacts with FAD2. ALA10 expression affects PC fatty acyl desaturation by limiting FAD3 over FAD2 activity, thus enhancing the level of 18:2 containing PC and decreasing the level of 18:3 PC (Poulsen et al., 2015; Botella et al., 2016). ALA10 also interacts with a β-subunit, ALA-Interacting Subunit (ALIS), either ALIS1 or ALIS5, leading to a preferential endomembrane localization dependent on the interacting protein, close to the plasma membrane with ALIS1 or to chloroplasts with ALIS5 (Botella et al., 2016). In leaves, ALA10 improves MGDG level especially in response to treatment of plants with Galvestine-1 or to growth at low temperature (Botella et al., 2016; Nintemann et al., 2019). It has been proposed that this positive effect operates *via* the activation of MGD1 by PA since it was neither associated with overexpression of MGD nor with enhancement of feeding of DAG coming from PC.

Supporting a regulation role of ALA10 in response to environmental modification, *ALA10* expression is highly variable and the protein very sensitive to degradation (Botella et al., 2016). One peptide of ALA10 had been previously detected in the proteome of plantlets treated with the 26S proteasome inhibitor, MG132, (Maor et al., 2007; Manzano et al., 2008) and prepared by ubiquitin affinity purification (Manzano et al., 2008). Although the ubiquitination of this peptide was not detected, this suggests a possible regulation of ALA10 by ubiquitination.

Ubiquitination may have several functions extending from protein targeting to degradation by either 26S proteasome system or vesicular trafficking to lytic compartments, to modification of activity and modification of protein molecular surroundings (Guerra and Callis, 2012). In plants, roles in regulation of nutrient import, in dynamics of endosymbiotic organelles and in hormone-mediated signaling have been further analyzed (Barberon et al., 2011; Guerra and Callis, 2012; Ling et al., 2012; Jung et al., 2015). Several works are based on characterization of E3 protein-ubiquitin ligases [for reviews see (Ling and Jarvis, 2013; Sharma et al., 2016; Trujillo, 2018)]. Two closely related proteins of the Plant U-box protein family, PUB10 and PUB11, have been recently studied to investigate their role in plant signaling (Jung et al., 2015). Both proteins are able to auto-ubiquitinate *in vitro* and to ubiquitinate several MYC family transcription factors, most importantly MYC2, a key regulator of jasmonic acid (JA) signaling. In addition, only PUB10, but not PUB11, played a role in regulation of JA signaling.

Here, we investigated the role of PUB11 in ALA10 post-translational modification. We show the interaction of ALA10 with PUB11 and ubiquitination of ALA10. The function of ALA10 was investigated in relation with its impact on PC and MGDG metabolism in Arabidopsis leaves.

## Material and Methods

### Plant Materials and Growth Conditions

The *Arabidopsis thaliana ala10* and *pub11* lines, respectively SALK_024877 for insertion in At3g25610 and SALK_029828 for At1g23030, are in *Col-0* background and came from the SALK institute collection (Alonso et al., 2003). Characterization of the *ala10* mutant line was previously described in (Botella et al., 2016). The *pub11* line was previously reported in (Jung et al., 2015) and homozygous insertion was verified in this work by PCR analysis on genomic DNA using the T-DNA primer (LB, GGCAATCAGCTGTTGCCCGTCTCACTGGTG) and the gene-specific primer (PUB11Rv2, GAT TCT CTT CGC AGC ACC AT) for detection of the interrupted copy of the gene and two gene-specific primers (PUB11Rv2, GAT TCT CTT CGC AGC ACC AT and PUB11Fw3, AGA ACC AAT CCC AAA GCT CA) for control of WT copy.

Construction of L1 and L2 lines with ALA10-GFP expression in *Col-0* background is described in (Botella et al., 2016). For expression of ALA10-GFP expression in the *pub11* background, ALA10-GFP was obtained by PCR-amplification from an *Arabidopsis* cDNA library using ALA10 flanking primers (ALA10F2, ATGGCTGGTCCAAGTCGGAGAAGAAG and ALA10R2, TTAGACACCGACAAGATCCTTATAGATCTGATCGTG) and the cDNA fragment was cloned in pUC18 upstream of the GFP S65T cDNA and under the control of the cauliflower mosaic virus 35S promoter and the *nos* terminator. Construction was transferred into the pEL103 binary vector for *Agrobacterium tumefaciens* transformation. For expression of GFP-ALA10 expression in WT and *pub11* background, GFP-ALA10 construction in the pMDC45 binary plasmid was a gift from Rosa L. Lopez-Marques and described in Poulsen et al. (2015). *Arabidopsis* plants were transformed by floral dipping (Mittler and Lam, 1995; Clough and Bent, 1998). Transformants were selected by several rounds of selection on MS medium containing 50 µM kanamycin for ALA10-GFP and 25 mg/ml^–1^ hygromycin solution for GFP-ALA10 until the isolated line shows at least 85% of resistant plants.

In standard culture, plants were grown for 2 weeks on solid MS medium containing 0.5% sucrose, 0.8% agar, 0.5 g L^−1^ MES/KOH pH 5.7. In liquid culture, plants were grown for 10 days on the same medium without agar. Ten seeds were sawn in 1.5 ml medium in each well of a 6 well-plate. After 2 days of stratification at 4°C, plates were placed in a growth chamber at 20°C with white light 100 μmol m^−2^ s^−1^ under long-day (16-h light/8-h dark) conditions. Treatment of plants with MG132 was done on liquid culture by removing the medium and replacing with a new medium supplemented with 50 µM of MG132 prepared in DMSO or 0.1% DMSO for control. Medium replacement was done at 4:00 pm on day 9 and plant harvest at 1:00 pm on day 10. Plants were immediately frozen in liquid nitrogen and kept at −80°C before analysis.

### Membrane Preparation

For protein purification, membranes were prepared as follows: frozen tissues were ground to powder in liquid nitrogen. Approximately 300 µl of thawed powder was homogenized in 1.5 ml of ice cold grinding medium (15 mM MOPS/NaOH pH 7.5, 2 mM EGTA, 0.6% w/v polyvinylpyrrolidone 25, 10 mM DTT, 1mM phenylmethylsulfonyl fluoride, 1mM benzamidine, 5 mM caproic acid, 5 mM iodoacetamide, 50 µM PR619, 10 µM MG132, 0.2% DMSO). Supernatant was then collected by microcentrifugation of the suspension at 400×*g* for 10 min at 4°C before microcentrifugation at 10,000×*g* for 10 min at 4°C. Membranes were collected in pellets.

### Protein Immunopurification

Five membrane pellets corresponding to a total of 0.5 mg protein were homogenized in 0.75 ml of solubilization buffer (50 mM imidazole pH7.5, 500 mM 6-aminocaproic acid, 1 mM EDTA, 1% Triton X100). Mixture was incubated for 30 min on ice before centrifugation at 100,000×*g* for 20 min at 4°C (Hitashi microcentrifuge). Supernatant was collected and 45 µl withdrawn for control of column input. 50 µL of anti-GFP µMACS magnetic beads were added to approximately 700 µl supernatant and incubated for 1h on ice. Mixture was then loaded on prewashed µMACS column set on a magnetic support (Milteniy Biotech). The column was washed with 400 µl of solubilization buffer, 1.6 ml of solubilization buffer containing 0.1% Triton X100 instead of 1%, then 150 µl of 20 mM Tris–HCl pH 7.5. Proteins were then eluted with 95 µl of Miltenyi denaturing elution buffer at 95°C and kept at −80°C before analysis.

### Protein Immunodetection

Protein was resolved by Laemmli SDS-PAGE on 4–15% polyacrylamide gel before electrotransfer to 0.2 µm nitrocellulose membrane in Laemmli buffer, 20% ethanol, 0.02% SDS. Protein transfer was controlled by Ponceau red staining of the membrane and positioned with Precision Plus Protein Dual Color standards (Eurogentec). The membranes were blocked in TBS, 0.1% Tween-20, 5% BSA. Immunostaining was done by incubation of the blot with specific antibodies in TBS, 0.1% Tween-20, 1% BSA, washing in TBS, 0.1% Tween-20 and detection by horseradish-peroxidase coupled reaction visualized on Chemidoc MP Imaging system (BioRad). Antibodies against ALA10 were obtained by rabbit immunization with the ALA10 C-terminus peptide RSARFHDQIYKDLVGV fused to the N-terminus of ovalbumin and affinity purification on the peptide. Antibodies were used at the following dilution: anti-ALA10 at 1/10,000, anti-GFP-HRP conjugated (Mitenyi Biotech) at 1/5,000, anti-ubiquitin (P4D1)-HRP conjugated (Cell signaling) at 1/1,000, anti-ubiquitin Lys48-specific (Millipore) at 1/1,000, anti-ubiquitin Lys63-specific (Millipore) at 1/1,000, anti SMT (Agrisera) at 1/5,000.

### Proteomic Analysis by Mass Spectrometry

#### Protein Digestion

Proteins immunopurified from the L2 plant extract were run on a SDS-PAGE gel. The bands corresponding to the ALA10-GFP protein were manually excised for in-gel digestion with trypsin using a Freedom EVO150 robotic platform (Tecan Traging AG, Switzerland) as follows. Gel bands were washed six times by successive incubations in 25 mM NH_4_HCO_3_ and then in 50% (v/v) CH_3_CN, 25 mM NH_4_HCO_3_. After dehydration in pure CH_3_CN, reduction was carried out with 10 mM DTT in 25 mM NH_4_HCO_3_ (45 min at 53°C) and alkylation with 55 mM iodoacetamide in 25 mM NH_4_HCO_3_ (35 min in the dark). Alkylation was stopped by the addition of 10 mM DTT in 25 mm NH_4_HCO_3_ (10-min incubation). Gel pieces were then washed again in 25 mM NH_4_HCO_3_ and dehydrated with pure acetonitrile. Modified trypsin (sequencing grade, Promega) in 25 mM NH_4_HCO_3_ was added to the dehydrated gel pieces for incubation at 37°C overnight. Peptides were extracted from gel pieces in three sequential extraction steps (each 15 min) in 30 μl of 50% (v/v) CH_3_CN, 30 μl of 5% (v/v) formic acid, and finally 30 μl of pure CH_3_CN. The pooled supernatants were dried under vacuum.

#### Nano-LC–MS/MS Analyses

The dried extracted peptides were resuspended in 5% acetonitrile and 0.1% trifluoroacetic acid and analyzed *via* online nano-LC–MS/MS (Ultimate 3000 RSLCnano and Q-Exactive Plus, Thermo Fisher Scientific). Peptide mixtures were desalted on line using a reverse phase precolumn (Acclaim PepMap 100 C18, 5 μm bead size, 100 Å pore size, 5 mm × 300 μm, Thermo Fisher Scientific) and resolved on a C18 column (Reprosil-Pur 120 C18-AQ, 1.9 μm, 25 cm × 75 μm, Dr. Maisch HPLC GmbH). The nano-LC method consisted of a 60 min multi-linear gradient at a flow rate of 300 nl/min, ranging from 5 to 33% acetonitrile in 0.1% formic acid. Spray voltage was set at 1.5 kV and heated capillary was adjusted to 250°C. Survey full-scan MS spectra (m/z = 400–1600) were acquired with a resolution of 70,000, with AGC target set to 10^6^ ions (maximum filling time 250 ms) and with lock mass option activated. The 10 most intense ions were fragmented by higher-energy collisional dissociation (nce = 30) with a resolution of 17,500, with AGC target set to 10^6^ ions (maximum filling time 250 ms and minimum AGC target of 3 × 10^3^), and dynamic exclusion set to 20 s. MS and MS/MS data were acquired using the Xcalibur software (Thermo Scientific).

#### Database Searches and Results Processing

Data were processed automatically using Mascot Distiller software (version 2.6, Matrix Science). Peptides and proteins were identified using Mascot (version 2.6, Matrix Science) through concomitant searches against TAIR (version 10.0), classical contaminants database (homemade), and their corresponding reversed databases. Trypsin/P was chosen as the enzyme and three missed cleavages were allowed. Precursor and fragment mass error tolerance were set, respectively, to 10 ppm and 25 mmu. Variable peptide modifications allowed during the search were: carbamidomethylation (C), acetyl (Protein N-ter), oxidation (M), and diGlycine (K). The Proline software (http://proline.profiproteomics.fr) was used to filter the results: conservation of rank 1 peptide-spectrum match (PSM) with a minimal length of 7 and a minimal score of 25. PSM score filtering was used to reach a False Discovery Rate (FDR) of PSM identification below 1% by employing the target-decoy approach. The mass spectrometry proteomics data have been deposited to the ProteomeXchange Consortium *via* the PRIDE (Perez-Riverol et al., 2019) partner repository with the dataset identifier PXD019412 and 10.6019/PXD019412”.

### Glycerolipid Analysis

Glycerolipids were extracted from approximately 150 mg of fresh material according to (Folch et al., 1951). Tissue were frozen in liquid nitrogen immediately after harvest, freeze-dried and ground in 4 ml of boiling ethanol for 5 min followed by addition of 2 ml of methanol and 8 ml of chloroform. After saturation with argon and filtration through glass wool, remains were rinsed with 3 ml of chloroform/methanol 2:1, v/v and lipids further extracted by addition of 5 ml of NaCl 1%. The chloroform phase was dried under argon before solubilization in pure chloroform and analysis of fatty acid content. For FA analysis, FAs were methylated using 3 ml of 2.5% H_2_SO_4_ in methanol during 1 h at 100°C (including standard amounts of 21:0). The reaction was stopped by the addition of 3 ml of water and 3 ml of hexane. The hexane phase was analyzed by gas liquid chromatography (Perkin Elmer) on a BPX70 (SGE) column and detected by flame ionization detector FID. Methylated fatty acids were identified by comparison of their retention times with those of standards and quantified by surface peak method using 21:0 for calibration. Lipids were analyzed by LC–MS/MS as described by (Jouhet et al., 2017). The lipid extracts corresponding to 25 nmol of total FA were dissolved in 100 μl of chloroform/methanol [2/1, (v/v)] containing 125 pmol of each of the three internal standards, DAG 18:0–22:6, PE 18:0–18:0, and SQDG 16:0–18:0. HPLC separation was performed on an Agilent 1200 HPLC system using a 150 mm × 3 mm (length × internal diameter) 5 μm diol column (Macherey-Nagel). Mass spectrometric analysis was done on a 6460 triple quadrupole mass spectrometer (Agilent) equipped with a Jet stream electrospray ion source. For adjustment of lipid quantification, each sample was run in tandem with a lipid extract from a qualified control (QC) of *A. thaliana* leaves. Analysis was done on five biological replicates or as specified. Statistical relevance was based on a Student’s test or as indicated in the figure legend.

### Confocal Microscopy of ALA10-GFP Lines

Confocal imaging: Slides were observed with an oil immersion 40× objective at room temperature by confocal laser scanning microscopy using a Zeiss LSM 800. GFP and chlorophyll were excited and signal collected sequentially (line by line, two to eight lines average) using the 488 nm laser diode for GFP and the 640 nm laser diode for chlorophyll. Fluorescence was collected between 505 and 514 nm, and 658 and 700 nm for GFP and chlorophyll respectively. All images were acquired using the same acquisition parameters.

### BiFC Analysis

cDNAs encoding ALA10 and PUB11 were amplified by PCR and cloned in pENTR:D/TOPO (Invitrogen) using primers ALA10BiFCFw (CACCATGGCTGGTCCAAGTCGGAG) and ALA10BiFCRv (GACACCGACAAGATCCTTATAGATCTGATC) for ALA10, PUB11WTFw (CAACATGCCGGAATGTTCAAG) and PUB11WTRv (TGTCCTGCCGTTAATGTAACCTG) for PUB11. cDNAs were then transferred to pBiFP1 and pBiFP4 for fusion with YFP Nter and YFP Cter respectively. The constructs were coexpressed in *Arabidopsis* protoplasts as described in (Botella et al., 2016). 24 to 48 h after transfection, samples were observed by confocal microscopy with an immersion 40× objective at room temperature by confocal laser scanning microscopy using a TCS-SP2 operating system (Leica). Fluorescence signals were visualized with an excitation line at 488 nm and a 10 nm collection window (average line by line of 2) for XY λ scan or excitation line at 488 nm with a 422 to 432 nm collection window (average line by line of 8) for YFP and excitation line at 633 nm line with a 658 to 700 nm collection window for chlorophyll (Sequential collection, 400 Hz, line by line).

## Results

### ALA10 Interacts With PUB11

**Figure 1:**
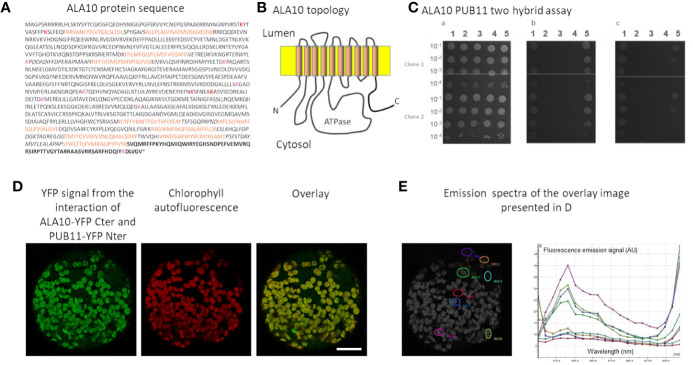
Analysis of ALA10-PUB11 interaction. **(A)** ALA10 protein sequence. In bold characters the C-ter fragment used in yeast two-hybrid screening and assays. Brown characters indicate the transmembrane fragments according to http://aramemnon.uni-koeln.de (Schwacke et al., 2003). Italics characters show the lumen facing protein portions. Lysine predicted with high and low confidence as ubiquitination sites for yeast or human proteins by the CKSAAP-UbSite (Chen et al., 2013) are indicated in red and pink, respectively. **(B)** Schematic topology of ALA10 in the ER membrane. **(C)** GAL4 Y2H two-hybrid interaction assay of ALA10 C terminal fragment with PUB11. Serial dilutions of cells were plated on non-selective or selective media. In **a**—growth on medium without tryptophane and leucine. In **b**—growth without tryptophane, leucine and histidine. In **c**- same condition as in b- with 1 mM of 3-aminotriazole. In column **1**—Smad3/Smurf1 positive control (Colland and Daviet, 2004), **2**—binding and activation domain alone negative control, **3**—binding domain alone and PUB11-activation domain, **4**—ALA10 Cter-binding domain and activation domain alone, **5**—ALA10 Cter-binding domain and PUB11-activation domain. **(D)** BiFC observation of ALA10-YFP Cter domain with PUB11-YFP Nter domain. Representative image over 20 observations. Protoplasts of Arabidopsis leaves were transfected with ALA10 and PUB11 fused respectively with YFP C ter and YFP N ter cloned in pBIFP1 and pBiFP4 and observed as assayed with combinations of ALA10 alone and ALA10 with ALISs in Botella et al. (2016). YFP signal is in green and Chlorophyll signal in red. Bar: 20 nm. **(E)** Emission spectra of the signal recorded in the YFP window at several positions of the image. Each position is indicated by a specific color.

In order to better understand ALA10 function, we sought protein partners by using a SPLIT-ubiquitin Arabidopsis protein library developed by Hybrigenics Service using the full-length ALA10 protein as a bait (MBmate screen, Hybrigenics Service). Since no interacting protein was identified in this first experiment due to an autoactivating effect of ALA10 construct in yeast, we used the yeast 2-hybrid system for screening and restrained the sequence of the bait to the soluble C-terminal extension of ALA10, that does not share homology with the other ALAs and is reported as topologically located on the cytosolic side of the ER membrane (**Figures 1A, B**) (ULTImate Y2H screen, Hybrigenics Service). Since ALIS subunits are supposed to interact with ALA transmembrane domain (Andersen et al., 2016), they are not expected to be found by using this strategy. PUB11 was obtained as the single very high confidence candidate interacting protein, with 12 independent yeast clones, each covering at least 40% of the protein sequence, including the N-terminus region. The interaction between the C-terminal peptide of ALA10 and the full length PUB11 was confirmed by subsequent 2-hybrid tests (**Figure 1C**) (1-by-1 Y2H Interaction Assay, Hybrigenics Service). To verify the interaction *in planta*, we used the BiFC (Bimolecular Fluorescence Complementation) technique. *Arabidopsis* leaf protoplasts were transfected with expression vectors for expression of full length ALA10 and PUB11 fused in C-ter with each complementary half of YFP. With both associations of YFP halves, YFP fluorescence was observed surrounding chloroplasts. YFP reconstitution indicated the proximity of ALA10 and PUB11 *in vivo* and suggested an interaction between the expressed proteins in plant cells (**Figures 1D, E** and **Supplementary Figure 1**). Considering that the sequence of PUB11 is typical for E3 protein ligases with a U box motif (Andersen et al., 2004; Trujillo, 2018), we investigated ALA10 ubiquitination.

### ALA10 Ubiquitination

**Figure 2:**
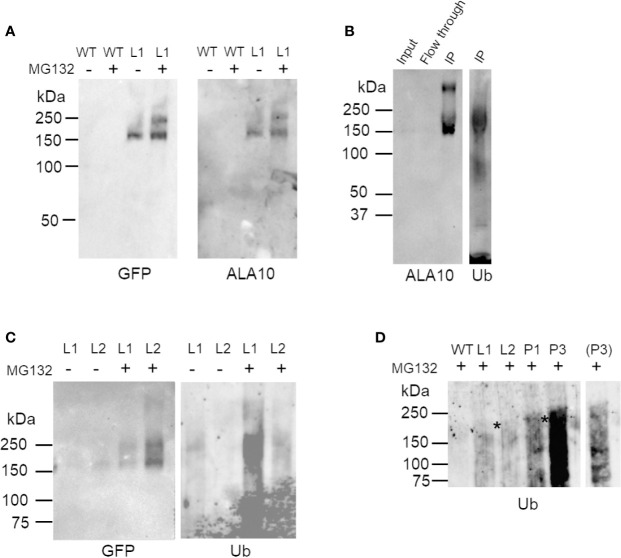
Western blot analysis of ALA10 ubiquitination. **(A)** Immunopurification of ALA10-GFP from plants with stable expression of ALA10-GFP (L1). Comparison with WT. Proteins are prepared from membranes extracted from plants treated the day before harvest with 50 µM MG132, 0.1% DMSO (+) or with only DMSO (−). They are solubilized with 1% Triton X100 and loaded on µMACS microbeads conjugated to an anti-GFP monoclonal antibody (Miltenyi). Purified fraction is analyzed by western blot with antibodies against ALA10 or GFP. **(B)**- ALA10-GFP fusion protein stably expressed in L2 plants was immunopurified (IP) on µMACS microbeads conjugated to an anti-GFP monoclonal antibody (Miltenyi) and analyzed by Western blot with anti-ALA10 and anti-ubiquitin antibodies. Plants were treated the day before harvest with 50 µM MG132 in 0.1% DMSO. **(C)** Immunopurification of ALA10-GFP from plants pretreated or not with MG132, an inhibitor of the proteasome. Samples are two lines of plants with different level of expression of ALA10-GFP, L1 and L2. Plants were treated the day before harvest with 50 µM MG132, 0.1% DMSO (+) or with only DMSO for control (−). Samples are analyzed with anti-GFP and anti-ubiquitin antibodies. **(D)** Comparison of ALA10 ubiquitination in *pub11* relative to WT. ALA10-GFP was immunopurified in two lines of *pub11* with stable expression of ALA10-GFP, P1 and P3, and compared to WT, L1 and L2. Plants were treated the day before harvest with 50 µM MG132, 0.1% DMSO. A shorter exposure of P3 lane is shown on the side. Stars indicate position of the highest ubiquitin signals that were detected in the L or P lanes.

Several putative sites of ubiquitination were predicted in the ALA10 sequence [**Figure 1A**, CKSAAP-Ubsite (Chen et al., 2011; Chen et al., 2013)]. To verify ALA10 ubiquitination, we purified the protein. For this, we started from ALA10-GFP overexpressing plant lines [called L1 and L2 (Botella et al., 2016)]. The ALA10-GFP fusion protein was purified from 10-day plantlets by GFP-affinity and analyzed by Western blot with anti-ubiquitin antibodies (**Figures 2A, B**). A signal, though prone to quick degradation, was obtained after addition in the medium of iodoacetamide and of the desubiquitinase inhibitor PR619. The signal was diffuse, visible above the position of ALA10-GFP (164 kDa) with an optimum around 200 kDa suggesting addition of five or six ubiquitins (**Figure 2B**). The purified ALA10-GFP was more abundant in samples prepared from plants treated with MG132, a proteasome inhibitor, compared to MG132 free controls and a similar increase of the ubiquitin signal was observed (**Figures 2A, C**). However, the ubiquitin signal was stronger in L1 line than in L2 even though ALA10-GFP was detected in lower amount in L1. In support of ALA10 ubiquitination, the immunopurified band corresponding to the L2 ALA10-GFP was analyzed by mass spectrometry and contained ubiquitin peptides. We characterized in this fraction between 28 and 50 ALA10 peptides covering around 30% of the protein, as well as GFP peptides (from 6 to 11) and ubiquitin peptides (from 1 to 4) (**Supplementary Table 1**). A few other *Arabidopsis* proteins were detected but not in all repeated experiments. We tried to identify ubiquitinated ALA10 peptides by searching diGly mark as described in (van der Wal et al., 2018). However, we could not identify any ubiquitinated peptide of ALA10 or of any other proteins, possibly due to a partial ubiquitination of proteins or a lack of abundance and ionization of the ubiquitinated peptides. Altogether, although we could not determine its ubiquitination sites, these data suggested that ALA10 is ubiquitinated. In addition, since MG132 treatment lead to higher levels of ALA10GFP, ALA10 ubiquitination could lead to its degradation by 26S proteasome.

### ALA10 Protein Level Is Sensitive to 26S Proteasome Degradation

**Figure 3:**
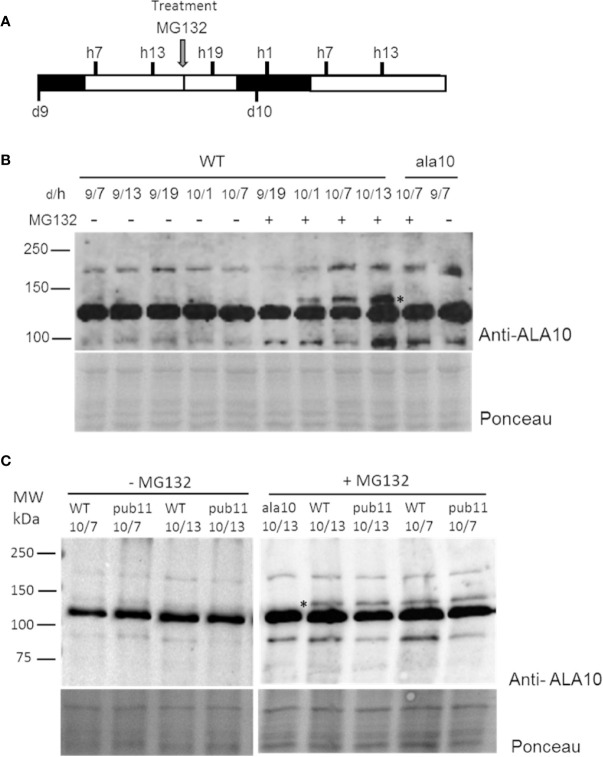
Analysis of ALA10 protein level at different times along days 9 and 10 of culture treated with MG132. **(A)** Time schedule of plantlet collection. Plantlets were grown hydroponically with a 16 h light/8 h dark photoperiod. They were treated at day 9, hour 16 by transfer to a medium containing 50 µM MG132, 0.1% DMSO (+). As a control (−), plantlets were transferred into the same medium containing only 0.1% DMSO. Plantlets were collected at hour 7, 13 and 19 of day 9 (9/h) and at hour 1, 7 and 13 of day 10 (10/h) and immediately frozen in liquid nitrogen. **(B)** Western blot analysis with ALA10 antibodies of WT and *ala10* membrane proteins of plantlets treated or not with MG132. **(C)** Western blot comparison of WT and *pub11* with ALA10 antibodies. A star indicates ALA10 position identified by comparison of WT and *ala10*. Ponceau staining is used for loading control.

In order to confirm this possibility and to understand the biological relevance of this degradation, we analyzed the expression profile of the ALA10 protein *in vivo*. We set up a western blot analysis of ALA10 at different times along the day in membrane extracts of 9- and 10-day old rosettes (**Figure 3A**), using the *ala10* mutant described in (Botella et al., 2016) as a control for detection of ALA10. ALA10 was detected as a weak signal above a major nonspecific signal at 130 kDa (**Figure 3B**), but we could not observe any difference of expression along the day in normal conditions. We observed that pretreatment of plants with MG132 increased ALA10 levels, supporting a degradation of ALA10 by 26S proteasome. We then checked whether ALA10 levels were modified in plants deprived of PUB11. An insertion mutant of the SALK collection was selected for a deletion of the pub11 gene (**Supplementary Figures 2A, B**) (Jung et al., 2015). In this mutant ALA10 levels looked relatively similar to those in the WT, with a similar increase after MG132 treatment (**Figure 3C**). Consequently, PUB11 did not appear critical for the degradation of ALA10 by 26S proteasome or for other process affecting protein content in presence of MG132.

In order to determine whether suppression of PUB11 coordinated or not with suppression of ALA10 ubiquitination, we transformed the *pub11* mutant to obtain stable expression of the ALA10-GFP fusion protein as previously done for L1 and L2 in the WT background. We wanted to analyze the ubiquitination of the ALA10-GFP fusion protein immunopurified from the transformed plants but the immunopurification of ALA10-GFP was not successful from the two independent lines. We then treated the plants with MG132 in order to increase ALA10-GFP content and prevent protein degradation. In this condition, in the two *pub11* ALA10-GFP lines (P1 and P3) we analyzed, we observed by western blot an ubiquitin signal in the immunopurified ALA10-GFP fraction that appeared stronger and different from those observed in the PUB11 ALA10-GFP lines, i.e. L1 and L2 (**Figure 2D**). In the P1 and P3 lines, the ubiquitin signal was diffuse with an optimum rather close to 240 kDa, consistent with the addition of eight or nine ubiquitins but with also some signals below 150 kDa, suggesting some degradation of ALA10. This indicated that ALA10 was still ubiquitinated in the absence of PUB11, but with a different pattern. Thus, PUB11 did not appear to catalyze the ubiquitination of ALA10 necessary for its degradation by 26S proteasome but nevertheless played a role in its ubiquitination pattern, its absence enhancing a distinct type of ALA10 ubiquitination.

### ALA10 Accumulates in Chloroplast-Associated Membranes in the Absence of PUB11

**Figure 4:**
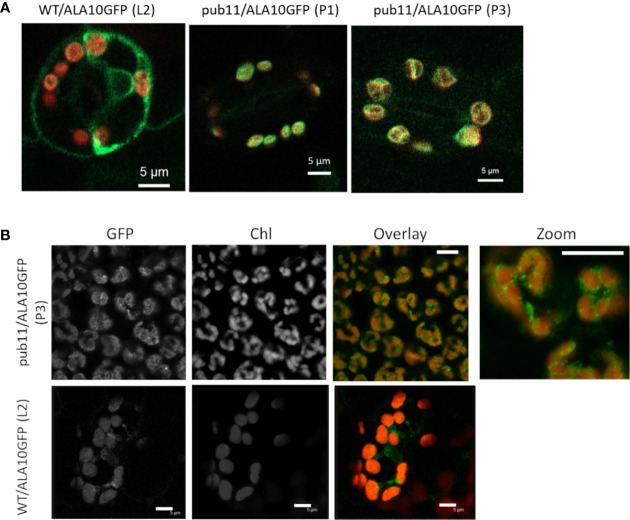
Localization of ALA10 in the *pub11* mutant by confocal microscopy. **(A)** and **(B)** Confocal imaging in WT or in *pub11* background with stable expression of ALA10-GFP in guard cells **(A)** and in mesophyll cells **(B)**. In **(A)** overlay of the signals in the GFP (green) and chlorophyll (red) windows. Observation in two different lines compared with WT. In **(B)**, observation in mesophyll cells of a line of *pub11* with stable expression of ALA10-GFP. Scale 5 µm.

Another major effect of protein ubiquitination is modification of the protein localization, which can affect both fate and activity of the protein (Barberon et al., 2011; Guerra and Callis, 2012). It was previously reported that ALA10 can display different localizations in the ER of mesophyll cells, with accumulation either next to the plasma membrane or next to the chloroplasts (Botella et al., 2016). In order to localize the protein in plants missing PUB11, we analyzed by confocal imaging the *pub11* mutants with stable expression of the ALA10-GFP fusion protein. In guard cells of both *pub11* lines exhibiting ALA10-GFP expression, we observed the GFP signal mainly around the chloroplasts, whereas in the WT background control, GFP was stronger at the periphery of the cell and at typical position for ER imaging such as around the nucleus (**Figure 4A**). The same observation was done in mesophyll cells (**Figure 4B**). Moreover, a similar difference was observed between WT and *pub11* lines with the GFP-ALA10 fusion protein holding GFP at the Cter of ALA10 instead at the Nter (**Supplementary Figure 3**). Based on these observations, PUB11 was necessary for the localization of ALA10 in a portion of the ER membrane non-associated with chloroplasts.

### Suppression of PUB11 Influences the Positive Effect of ALA10 on 18:3 Containing PC and MGDG Synthesis

**Figure 5:**
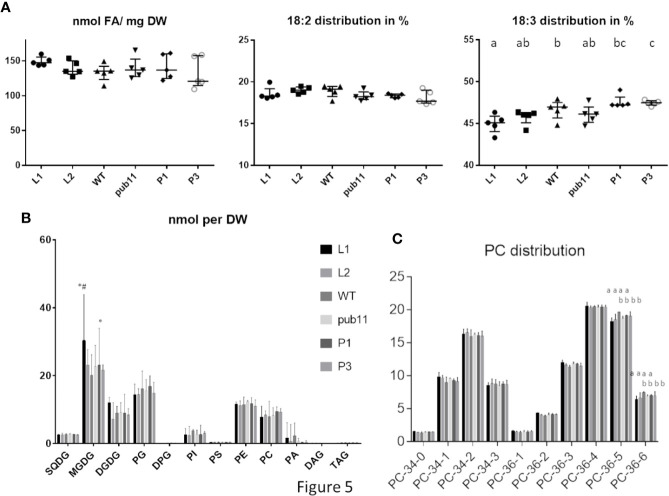
Lipid composition of aerial part of 15 d old plantlets of *pub11* lines overexpressing ALA10-GFP. **(A)** Fatty acid content and distribution of 18:2 and 18:3 fatty acids. (n = 5, Unpaired t test with Welch’s correction, P <0.05, differences between means that share a letter are not statistically significant). **(B)** Glycerolipid content. Glycerolipids were analyzed by MS and all glycerolipid data are expressed in nmol as adjusted to a standard reference of *Arabidopsis* leaf lipid extract run in parallel. All contents are expressed per dry weight. Lines which are compared are WT, *pub11* and overexpressors of ALA10-GFP in WT (L1 and L2) and in *pub11* (P1 and P3). Lipid are analyzed by MS (Jouhet et al., 2017). N = 5. * and # indicate statistically significant differences (Student’s t test with Welch’s correction, P <0.05) with WT (*) and *pub11* (#). **(C)** Relative distribution of the main molecular species of PC. (n = 5, two way ANOVA corrected for multiple comparison for C, P <0.05, differences between means that share a letter are not statistically significant).

Previous report showed that ALA10had a positive effect on leaf development, by counteracting MGDG synthesis limitation (Botella et al., 2016). We previously showed that ALA10 modulates the MGDG/PC ratio possibly through MGD1 activation by PA. At the molecular level, ALA10 interacts with FAD2 in the ER, limiting PC desaturation to 18:2; therefore, chloroplastic PC is enriched in 18:2, resulting in an enhancement of MGDG synthesis. This effect on PC pools occurs in quantitatively minor membrane domains; it is therefore challenging to analyze it at the level of the whole cell lipid extract, and requires the analysis of large sets of plantlets. We here compared the lipid composition of leaves in plant lines, which express or not exogenous ALA10-GFP either in a WT or a *pub11* background. Aerial parts of 15 days old seedlings were analyzed. The dry weight of rosettes showed a slight increase in the *pub11*/ALA10-GFP lines (P1 and P3) and a slight decrease in one of the ALA10-GFP overexpressor lines (L1) (**Supplementary Figure 4A**). We did not detect a significant variation of fresh weigh relative to dry weight (**Supplementary Figure 4A**). Per dry weight, the fatty acid content variations between each lines were not statistically significant (**Figure 5A**). Similarly, no difference was observed for the lipid content, except in the L1 line, where we observe an increase of MGDG, consistent with the increase of MGDG/PC ratio in this line (**Figure 5B** and **Supplementary Figures 5A, C**) already noticed in (Botella et al., 2016). However, this increase was not statistically significant when expressing the lipid contents in mol% (**Supplementary Figure 5B**). We also noticed a very low level of PA in the *pub11* lines whereas this amount is highly variable in the WT and L1 and L2 lines.

In this context, whereas in proportion the profiles of lipid classes appear rather similar in all lines, the overall fatty acid proportion was slightly altered with an increase of 18:3 in *pub11*/ALA10-GFP (P1 and P3) and a decrease of 18:3 in WT/ALA10-GFP (L1 and L2) lines compared to non overexpressor lines (WT and *pub11*). This implies a dual role of ALA10 depending on PUB11 (**Figure 5A** and **Supplementary Figure 4B**). This difference of fatty acid was detectable only in two classes of lipids: PC and MGDG (**Figure 5C** and **Supplementary Figure 5C**). The 36:5 and 36:6 molecular species of PC, corresponding respectively to 18:2/18:3 and 18:3/18:3 molecules, were decreased in the L1 and L2 lines (**Figure 5C**). This effect was lost when ALA10 was overexpressed in the *pub11* mutant context. The MGDG composition was also modified with a decrease of 34:6 compensated by an increase of 36:6 in the L1 and L2 lines (**Supplementary Figure 5C**), indicating an increase of the eukaryotic pathway relative to the prokaryotic pathway. Because in the L1 lines, MGDG quantity was significantly higher than in the other lines, there was no decrease of the 34:X MGDG species (prokaryotic pathway) but rather an increase of the 36:X MGDG species (eukaryotic pathway) (**Supplementary Figure 5C**). Altogether, although these analyses are difficult to perform and interpret, results are consistent with the fact that in the ALA10 overexpressing lines there is less 18:3 PC and more eukaryotic MGDG but that this effect is lost when PUB11 is absent.

## Discussion

Our data show that ALA10 is a P_4_-type ATPase prone to degradation by 26S proteasome. The protein was previously found in two proteomes of young seedlings treated by MG132 (Maor et al., 2007; Manzano et al., 2008). We here observed a strong increase of ALA10 amounts when the plants were treated by MG132 notably the day after treatment. Since we detected ubiquitin associated with purified ALA10 and an increase of this ubiquitin association after MG132 treatment, our data suggest that ALA10 is ubiquitinated and that its ubiquitination can lead to its degradation by the 26S proteasome. Considering that PUB11 is an E3 ubiquitin ligase (Jung et al., 2015), the interaction of ALA10 with PUB11 which we showed by yeast 2H and *in vivo* BiFC also supports ALA10 ubiquitination. Yet, we could not verify that ALA10 is directly ubiquitinated by PUB11. Deletion of PUB11 did not suppress the increase of ALA10 concentration when plants were treated with MG132 therefore suggesting that ALA10 degradation by 26S proteasome is not directly related to the interaction of ALA10 with PUB11. Moreover, ALA10 ubiquitination was not suppressed in the *pub11* mutant and the pattern of ALA10 ubiquitination looked different in this mutant. Thus, our results rather support that there are several types of ubiquitination of ALA10 and that PUB11 plays a role in ubiquitination of ALA10 independently of its 26S proteasome degradation.

What is then the function of the PUB11-dependent ubiquitination of ALA10? Several possible functions of ubiquitin modification have already been reported in plants, from alteration of abundance to modification of localization and shift in interaction properties (Guerra and Callis, 2012; Xie et al., 2015). Here we show that PUB11 does not seem to have an impact on ALA10 abundance but rather influences ALA10 localization. In the absence of PUB11, ALA10 is mainly present in the vicinity of chloroplasts and reduced or absent in the ER not associated with chloroplasts. This indicates a role of PUB11 in ALA10 localization. We previously showed that ALA10 localizes in different domains of the ER depending on the β subunit it interacts with, near chloroplasts with ALIS5 and close to the plasma membrane with ALIS1 (Botella et al., 2016). Our interpretation is that PUB11-dependent ubiquitination of ALA10 is detrimental to the interaction of ALA10 with specifically ALIS5, and favorable to both its interaction with ALIS1 and its localization close to the plasma membrane. In the absence of PUB11, improved molecular accessibility of ALA10 to ALIS5 would therefore favor its localization next to chloroplasts.

In plants, several cases where ubiquitination modifies protein localization have been described, for instance ubiquitination of iron and boron translocators in the plasma membrane. These ubiquitinations have a role in reducing the translocator abundance at the cell surface, and consequently inhibiting the ion uptake that prevents its toxicity in the cell (Barberon et al., 2011; Kasai et al., 2011; Zelazny et al., 2011). By endocytosis of the ubiquitinated protein, the translocator is sorted from the plasma membrane to the lytic vacuole where it is degraded. Our results suggest a role somewhat different for PUB11-dependent ubiquitination of ALA10 since we observed that the main degradation of ALA10 is related to 26S proteasome activity and independent of PUB11. Since PUB11 primarily affected the amount of ALA10 next to chloroplasts, this suggests a role of PUB11 in the turnover of chloroplast-associated ALA10.

ALA10 is a phospholipid flippase acting mainly on PC (Poulsen et al., 2015; Jensen et al., 2017). In *Arabidopsis* leaves, ALA10 interacts with FAD2 in the ER leading to an imbalance of PC desaturation towards an increase of 18:2-containing PC (Botella et al., 2016). FA desaturations in the ER are limiting steps during cell growth (Mei et al., 2015). Because of the light-dependence of FA synthesis, the increase in desaturated FAs during the dark period suggests also a daylight limitation of FA desaturation mainly in the ER on PC (Browse et al., 1981; Sasaki et al., 1997). Considering that ALA10 interacts with FAD2 and prevents production of 18:3-containing PC, it is likely that ALA10 contributes to preservation of an 18:2 pool of PC to the detriment of further desaturation into 18:3-containing PC. The preserved pool of 18:2-containing PC could feed the eukaryotic pathway for synthesis of galactolipids since an overexpression of FAD3 was reported to increase 18:3-enriched PC and PE and decrease MGDG (Shah et al., 1997). Consistently, we observed that PC 36:6 was slightly affected in the WT/ALA10-GFP lines compared to the WT and that this effect was lost in the *pub11*/ALA10-GFP lines. Since PC 36:6 contains two 18:3 FA, this suggests that PUB11 could play a role in ALA10-dependent limitation of 18:3-containing PC, possibly through ubiquitination of ALA10 and subsequent localization change.

Furthermore, because PA production seems altered in all *pub11* mutant backgrounds, this might explain why the increase of MGDG synthesis is not conserved when ALA10-GFP is overexpressed in *pub11* background. The decrease of the eukaryotic pathway proportion in MGDG production as well as a potential decrease of PA in *pub11* mutants support what was described in (Botella et al., 2017) and recently found by (Karki et al., 2019) where the authors showed that different pools of PC, probably localized in different membrane domains, are involved upstream MGDG production.

This work unravels a new partner of plant flippases, an ubiquitin ligase. To better understand these new functions in the context of the plant membrane homeostasis, it is crucial to understand plant flippase regulations to provide insights into the cellular processes driven by these proteins. The elucidation of ER membrane domain composition at different location of the cell might be a key element to apprehend these lipid homeostasis regulation and adaptation.

## Data Availability Statement

The mass spectrometry proteomics data have been deposited to the ProteomeXchange Consortium *via* the PRIDE [1] partner repository with the dataset identifier PXD019412 and 10.6019/PXD019412.

## Author Contributions

JJ conceived the original screening, supervised and complemented the writing. CB performed part of the experiments. CA provided technical assistance to MB. JS performed part of the experiments. VG provided technical assistance to JJ. MB and JJ conceived the project, supervised the experiments, and wrote the article with contributions of all the authors.

## Funding

Authors were supported by LabEX GRAL, ANR-10-LABX-49-01 financed within the University Grenoble Alpes graduate school (Ecoles Universitaires de Recherche) CBH-EUR-GS (ANR-17-EURE-0003), by the French ANR through Glyco@Alps (ANR-15-IDEX-02) and Young Scientist grant ChloroMitoLipid (ANR–12–JSV2–0001).

## Conflict of Interest

The authors declare that the research was conducted in the absence of any commercial or financial relationships that could be construed as a potential conflict of interest.

## Supplementary Material

Supplemental Table 1 Result of mass spectrometry analysis of the immunopurified ALA10-GFP fraction purified on a SDS-PAGE gel between 150 and 200 kDa. Results are from 3 independent biological and immunopurification experiments. In purple is emphasized the peptides corresponding to ALA10 protein, in green to GFP and in yellow to the ubiquitin.
