# Supplementary material for: PUB11-Dependent Ubiquitination of the Phospholipid Flippase ALA10 Modifies ALA10 Localization and Affects the Pool of Linolenic Phosphatidylcholine
Source: Front Plant Sci. 2020 Jul 15;11:1070. doi: 10.3389/fpls.2020.01070 (PMC7373794; doi:10.3389/fpls.2020.01070)
Supplement: Supplementary file 2 [file Presentation_1.pptx]

## Slide 1
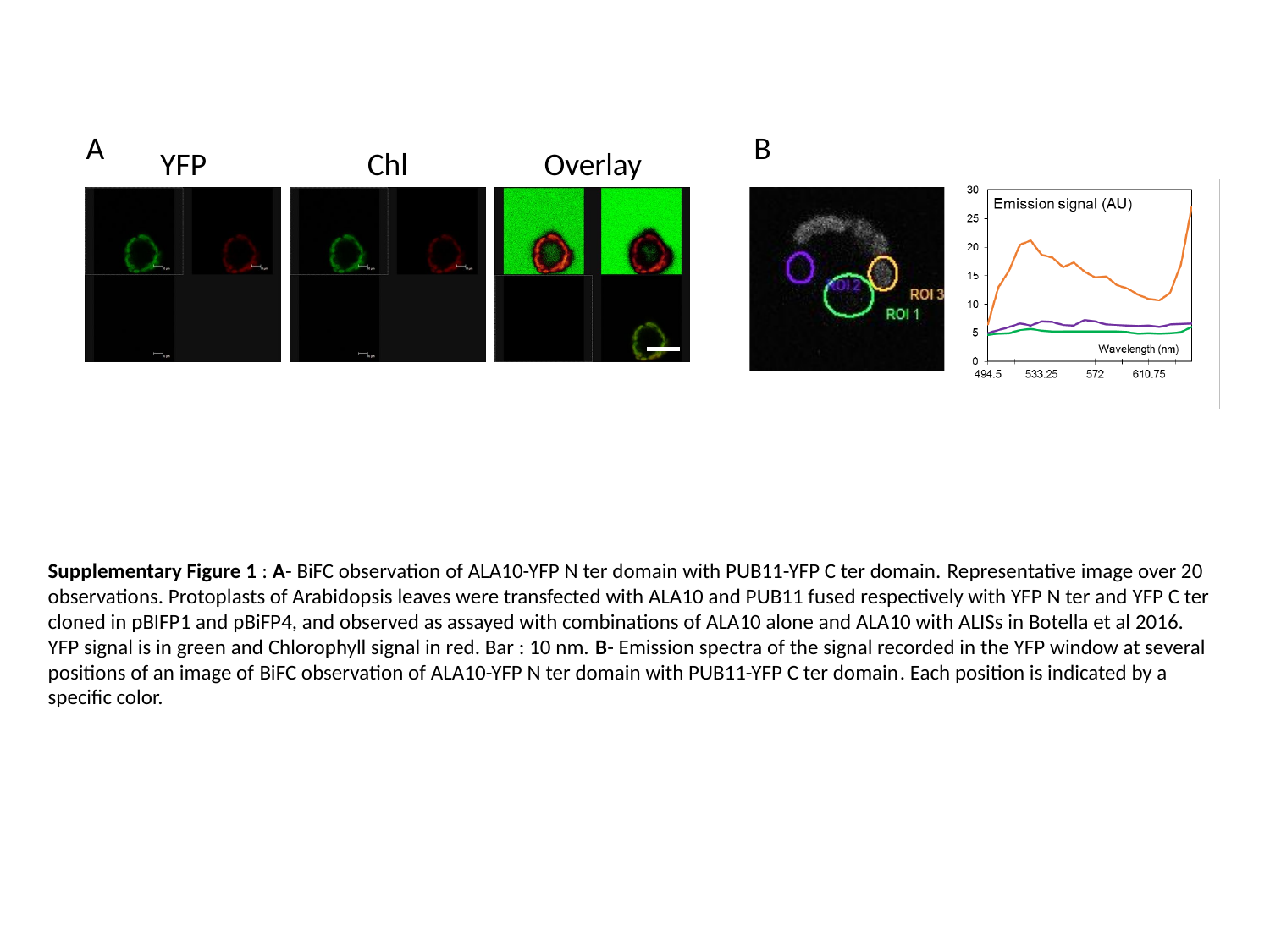

A
B
YFP
Chl
Overlay
Supplementary Figure 1 : A- BiFC observation of ALA10-YFP N ter domain with PUB11-YFP C ter domain. Representative image over 20 observations. Protoplasts of Arabidopsis leaves were transfected with ALA10 and PUB11 fused respectively with YFP N ter and YFP C ter cloned in pBIFP1 and pBiFP4, and observed as assayed with combinations of ALA10 alone and ALA10 with ALISs in Botella et al 2016. YFP signal is in green and Chlorophyll signal in red. Bar : 10 nm. B- Emission spectra of the signal recorded in the YFP window at several positions of an image of BiFC observation of ALA10-YFP N ter domain with PUB11-YFP C ter domain. Each position is indicated by a specific color.

## Slide 2
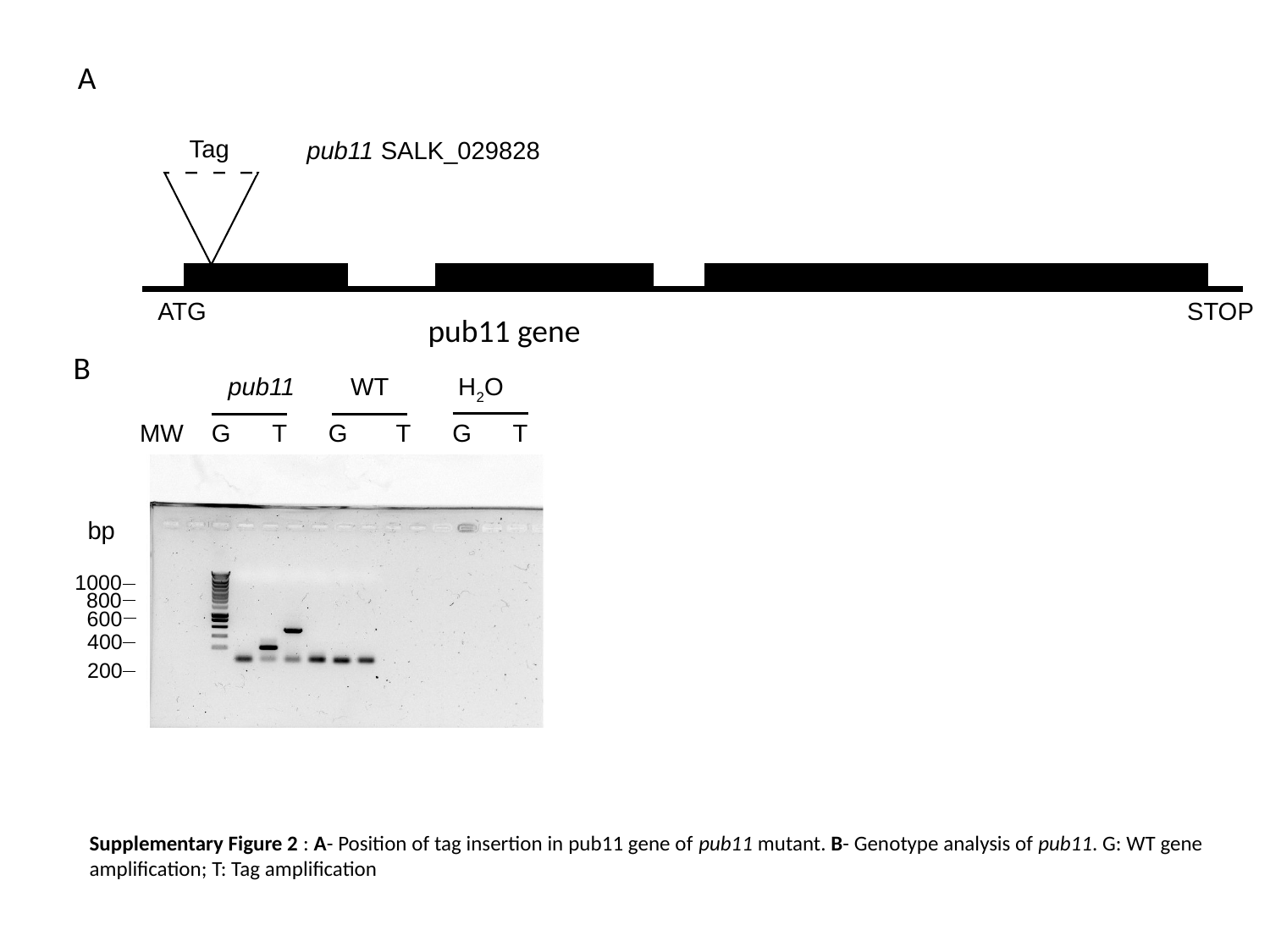

A
Tag
pub11 SALK_029828
pub11 WT H2O
ATG
STOP
pub11 gene
B
 MW G T G T G T
bp
1000
800
600
400
200
Supplementary Figure 2 : A- Position of tag insertion in pub11 gene of pub11 mutant. B- Genotype analysis of pub11. G: WT gene amplification; T: Tag amplification

## Slide 3
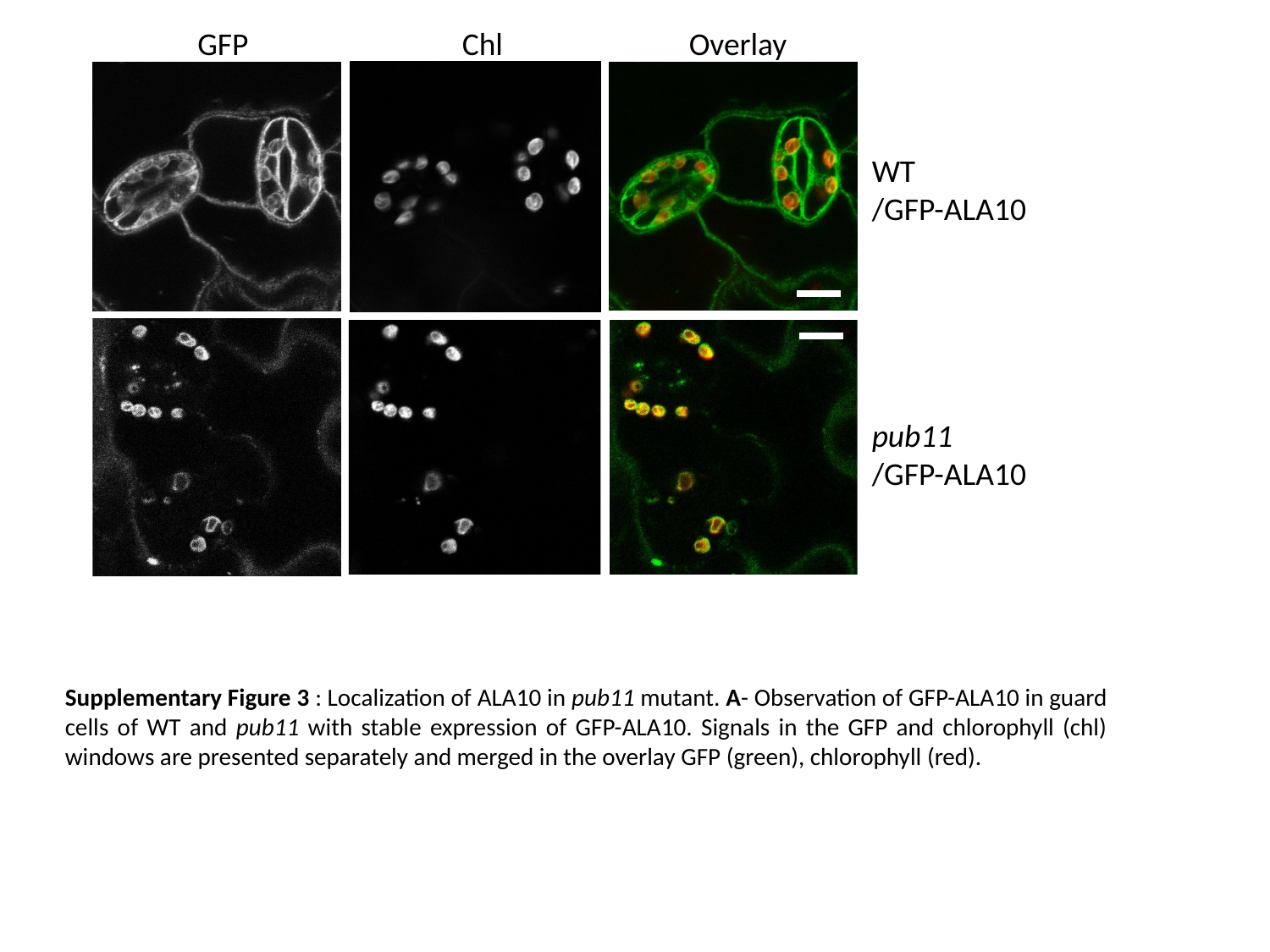

GFP
Chl
Overlay
WT
/GFP-ALA10
pub11
/GFP-ALA10
Supplementary Figure 3 : Localization of ALA10 in pub11 mutant. A- Observation of GFP-ALA10 in guard cells of WT and pub11 with stable expression of GFP-ALA10. Signals in the GFP and chlorophyll (chl) windows are presented separately and merged in the overlay GFP (green), chlorophyll (red).

## Slide 4
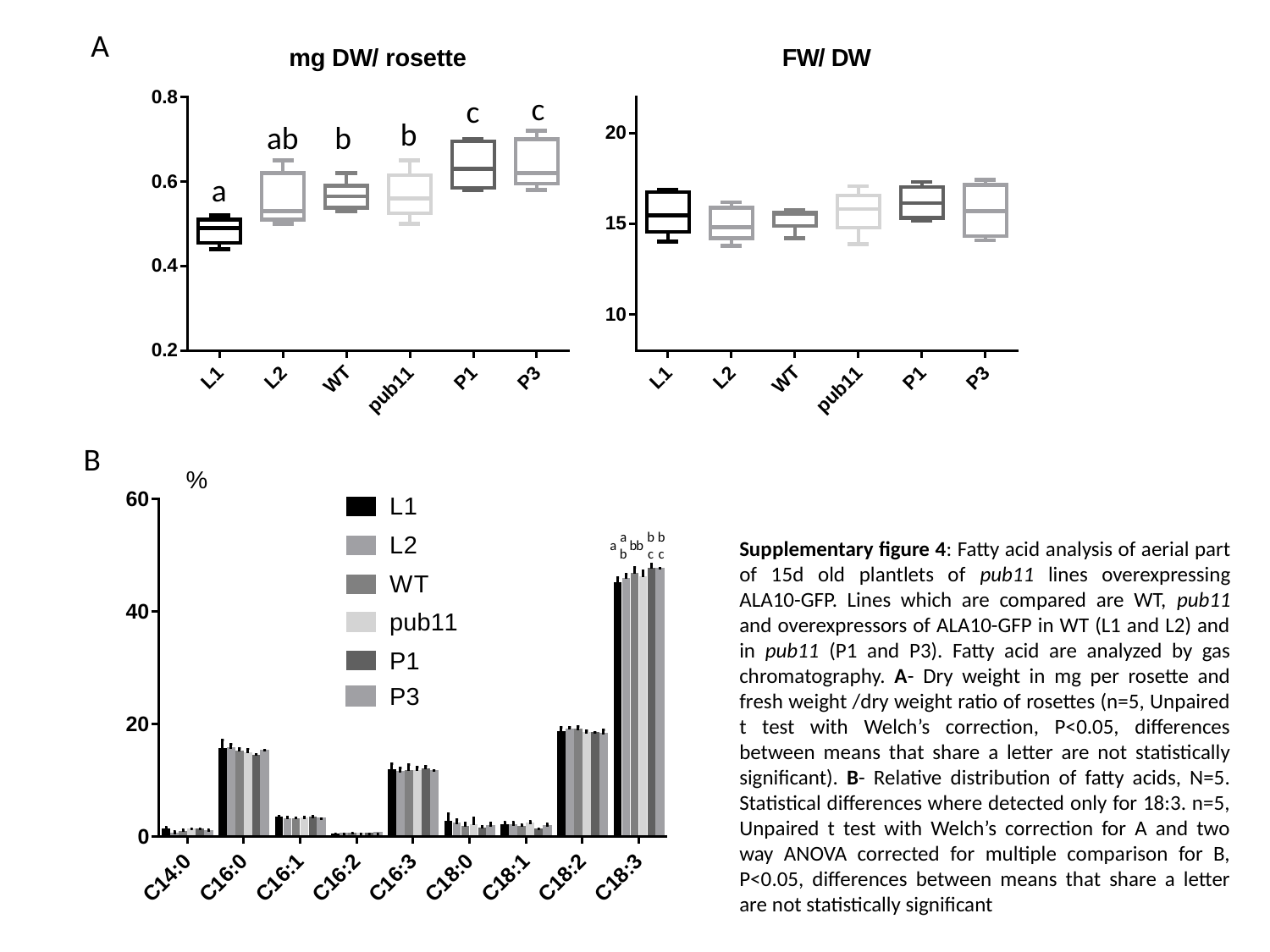

A
c
c
b
b
ab
a
B
ab
bc
bc
b
Supplementary figure 4: Fatty acid analysis of aerial part of 15d old plantlets of pub11 lines overexpressing ALA10-GFP. Lines which are compared are WT, pub11 and overexpressors of ALA10-GFP in WT (L1 and L2) and in pub11 (P1 and P3). Fatty acid are analyzed by gas chromatography. A- Dry weight in mg per rosette and fresh weight /dry weight ratio of rosettes (n=5, Unpaired t test with Welch’s correction, P<0.05, differences between means that share a letter are not statistically significant). B- Relative distribution of fatty acids, N=5. Statistical differences where detected only for 18:3. n=5, Unpaired t test with Welch’s correction for A and two way ANOVA corrected for multiple comparison for B, P<0.05, differences between means that share a letter are not statistically significant
a
b

## Slide 5
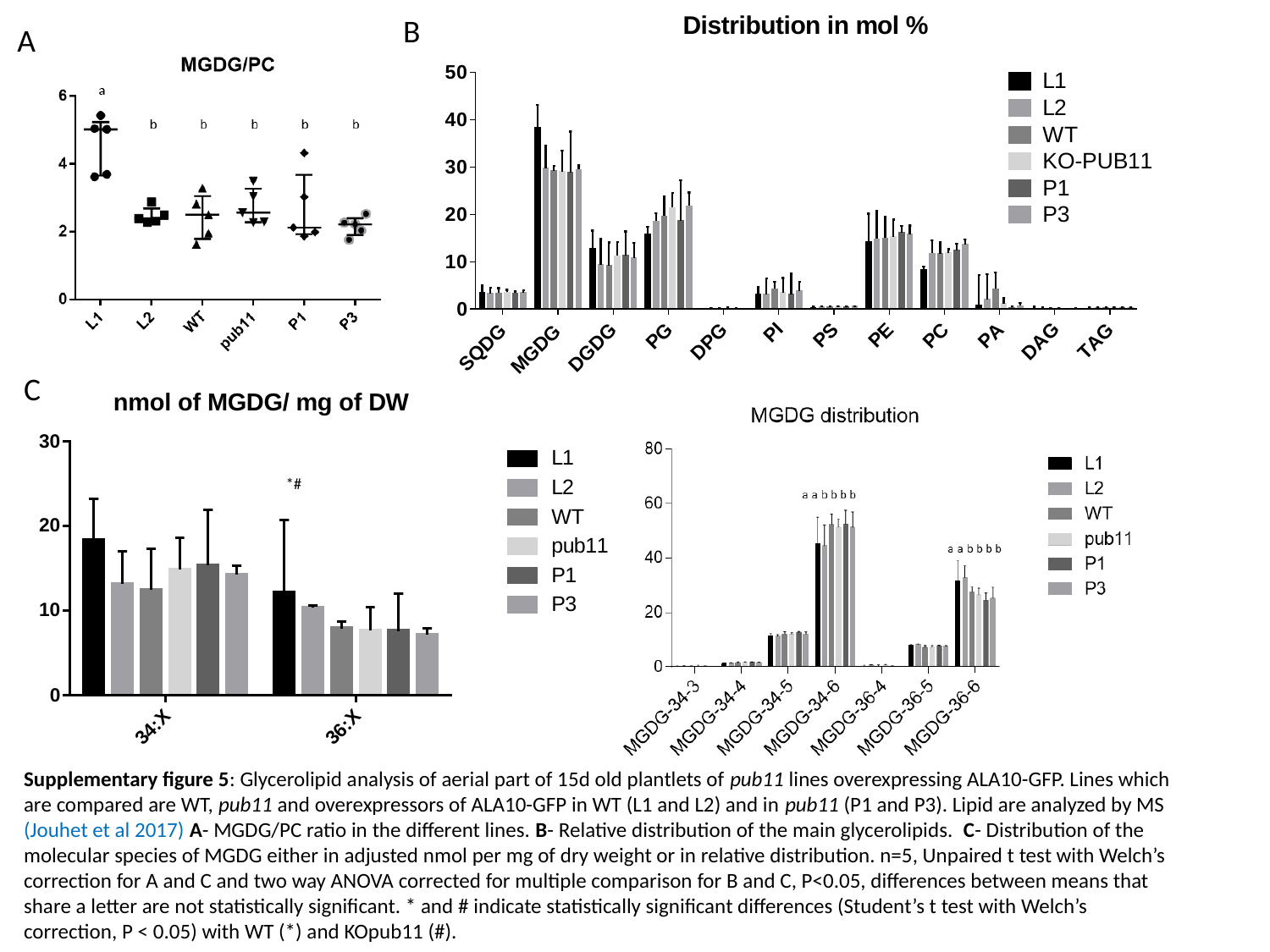

B
A
C
*#
Supplementary figure 5: Glycerolipid analysis of aerial part of 15d old plantlets of pub11 lines overexpressing ALA10-GFP. Lines which are compared are WT, pub11 and overexpressors of ALA10-GFP in WT (L1 and L2) and in pub11 (P1 and P3). Lipid are analyzed by MS (Jouhet et al 2017) A- MGDG/PC ratio in the different lines. B- Relative distribution of the main glycerolipids. C- Distribution of the molecular species of MGDG either in adjusted nmol per mg of dry weight or in relative distribution. n=5, Unpaired t test with Welch’s correction for A and C and two way ANOVA corrected for multiple comparison for B and C, P<0.05, differences between means that share a letter are not statistically significant. * and # indicate statistically significant differences (Student’s t test with Welch’s correction, P < 0.05) with WT (*) and KOpub11 (#).
